# Immersive Metaverse Learning Environment (IMLE) to Improve Social-Cognitive Skills in STEAM Learning in Elementary Schools

**DOI:** 10.12688/f1000research.172295.1

**Published:** 2025-12-22

**Authors:** Reza Rachmadtullah, Rasmatidila, Rufi'i Rufi'i, Hanim Faizah, Amelia Rizky Idhartono Idhartono, Cholifah Tur Rosidah

**Affiliations:** 1 Elementary Education, Universitas PGRI Adi Buana Surabaya, Surabaya, East Java, 60233, Indonesia; 2 Elementary Education, Universitas Djuanda, Bogor, West Java, 16720, Indonesia

**Keywords:** Immersive Metaverse Learning Environment (IMLE), social-cognitive skills, STEAM, elementary school, innovative learning

## Abstract

**Background:**

The development of social-cognitive skills is increasingly important in STEAM (Science, Technology, Engineering, Arts, and Mathematics) learning at the elementary school level. Immersive Metaverse Learning Environments (IMLEs) offer students the opportunity to learn in interactive virtual spaces, but research on their integration into STEAM learning remains limited, particularly in enhancing cognitive, social, and collaborative aspects.

**Purpose:**

This study aims to test the effectiveness of using IMLE technology in improving social-cognitive skills in STEAM learning in elementary schools.

**Design/Methodology/Approach:**

This study employed a mixed-methods design, combining quantitative and qualitative approaches. Quantitative data were used to assess the effectiveness of IMLE in improving social-cognitive skills, while qualitative data explored student and teacher experiences through observations, interviews, and analysis of classroom learning activities. The study subjects included 50 elementary school students in Surabaya, Indonesia.

**Results:**

The study findings showed significant improvements in students’ social-cognitive skills, including collaboration, problem-solving, empathy, communication, and learning motivation. Furthermore, the use of IMLE contributed to improved STEAM learning outcomes and enriched learning interactions.

**Originality/Value:**

Research on the integration of IMLE into STEAM learning in elementary schools is still rare, both in Indonesia and globally. This study demonstrates that IMLE effectively supports the development of social-cognitive skills while encouraging innovation in immersive technology-based learning practices. These findings provide valuable contributions to teachers in adopting interactive, student-centered learning media.

## Introduction

The development of digital technology in 21st-century education has presented new opportunities for creating more interactive, collaborative, and contextual learning. One innovation currently being widely discussed is the use of the Immersive Metaverse Learning Environment (IMLE) ( Yeganeh et al., 2025; Beck, Morgado, & O’Shea, 2024). This technology offers an immersive learning experience through virtual world simulations that enable students to interact, collaborate, and explore in a more realistic way. In the context of elementary education, particularly STEAM (Science, Technology, Engineering, Arts, and Mathematics) learning, IMLE is believed to provide a learning experience that emphasizes not only cognitive aspects but also fosters social-cognitive skills such as collaboration, self-regulation, empathy, and perspective-taking. In this context, the Science, Technology, Engineering, Arts, and Mathematics (STEAM) approach is considered an effective educational strategy for preparing students to face future challenges ( Singh, Sun, & Zheng, 2024; Natale et al., 2024).

Despite the current technological developments in education, teachers must be able to select and design appropriate learning technologies for use in classroom teaching and learning activities. A common problem faced by teachers today is the low level of student engagement and social-cognitive abilities in STEAM learning in elementary schools ( Chang, Wang, & Ku, 2023). STEAM learning, which should develop creativity, collaboration, and critical thinking, still tends to focus solely on cognitive aspects ( Buragohain, Meng & Chaudhary, 2025). Furthermore, students’ STEAM learning outcomes in Indonesia are still low because students have not been able to connect science and technology concepts with everyday life in a contextual and collaborative manner. STEAM learning should emphasize interdisciplinary integration and develop problem-solving skills, creativity, and innovation ( Zhao & Abdullah, 2025). However, the reality is that, the implementation of STEAM in elementary schools often faces limitations in terms of learning methods that effectively stimulate social-cognitive skills. Many learning practices still tend to be solely oriented toward cognitive outcomes, while aspects of collaboration, empathy, social perspective, and self-regulation have not received adequate attention ( Vadivu et al., 2025; Filipe, Baptista & Conceição, 2024). Along with technological advances, innovations have emerged in the form of Immersive Metaverse Learning Environments (IMLEs), which offer learning experiences based on virtual reality (VR), augmented reality (AR), and three-dimensional digital interactions ( Filipe, Baptista & Conceição, 2024). IMLEs enable students to explore learning content in an immersive, interactive, and collaborative space. Seiari, Kaabi & Al-Karaki (2023) consider this technology to have the potential to increase engagement, motivation, and social-cognitive skills, as students can interact virtually as if they were in a real community.

Recent research, as reported by Gaikwad et al. (2025) shows that metaverse environments can enhance collaboration between students and encourage deeper, experiential learning. However, the use of IMLE in elementary education still faces several obstacles. First, there is a lack of a structured implementation model tailored to the developmental characteristics of elementary school children. Second, many teachers lack sufficient technological literacy and pedagogical skills to integrate IMLE into daily learning practices. Third, limited empirical evidence regarding the effectiveness of IMLE in improving children’s social-cognitive skills remains a challenge, given that most research emphasizes motivation and learning experiences rather than measuring specific social and collaborative skill outcomes ( Lyu & Fridenfalk, 2024; Chamola et al., 2025). Furthermore, technical issues such as device availability, physical comfort, and issues of digital ethics and security also pose obstacles that need to be considered.

Several previous studies have shown that immersive technology, both in the form of Virtual Reality and Augmented Reality, can increase student engagement, the quality of the learning experience, and collaboration in STEAM activities. Diao & Su (2025) conducted an experimental study on elementary school students and found that using a metaverse environment in collaborative science experiments can enhance creativity and social interaction when designed based on principles of social-cognitive theory. Lee, Kim, and Byeon (2022) demonstrated that integrating metaverse learning into learning can enhance social presence, cognitive skills, and the development of 21st-century skills, although some studies suggest that the effects of enhanced experiences do not always translate directly to improved academic outcomes. Furthermore, a survey of elementary school teachers revealed a high level of interest in exploring the metaverse, but also emphasized the need for clearer training and pedagogical guidance ( Kaddoura & Husseiny, 2023).

Research on immersive technologies such as Virtual Reality (VR) and Augmented Reality (AR) has demonstrated significant improvements in student engagement and learning experiences. However, studies on Immersive Metaverse Learning Environments (IMLEs) in the context of STEAM learning in elementary schools are still rare. Most studies focus solely on motivation or social presence, rather than specific social-cognitive skills such as collaboration, empathy, communication, and self-regulation. Furthermore, research focuses primarily on secondary and tertiary levels, while the pedagogical needs of elementary schools have received little attention. This study aims to address this gap by examining the effectiveness of IMLEs in enhancing elementary school students’ social-cognitive skills in STEAM learning.

This research stems from a gap in the literature regarding the use of Immersive Metaverse Learning Environments (IMLE) in STEAM learning in elementary schools, particularly for developing students’ social-cognitive skills. Although immersive technology has been widely used to enhance interactive and realistic learning experiences, studies specifically examining the effectiveness of IMLE in developing collaboration, empathy, communication, and self-regulation in elementary school students are still very limited. This indicates the need for more in-depth research to fill this knowledge gap and enrich pedagogical practices in elementary education. Based on this background and research gap, the objectives of this study are: (1) To analyze the effectiveness of using IMLE in improving elementary school students’ social-cognitive skills in STEAM learning. (2) To explore students’ and teachers’ perceptions and experiences regarding the integration of IMLE into STEAM learning activities.

### Social-cognitive skills in STEAM learning in elementary schools

In Indonesia, the implementation of STEAM in elementary education still faces several obstacles, both in terms of curriculum and learning practices. The national curriculum still emphasizes basic literacy (reading, writing, and arithmetic), so dimensions of social-cognitive skills such as collaboration, communication, empathy, and self-regulation have not been prioritized in learning assessments. ( Guerrero, Valenciano-Valcárcel, & Rodríguez, 2024). Teachers are generally more focused on academic achievement, so the social-emotional aspect of teaching and learning is often neglected. Although local research is beginning to demonstrate the use of educational technology, including VR and AR, to increase student motivation, studies on the effectiveness of these technologies in supporting the development of social-cognitive skills are still very limited ( Onu, Pradhan, Mbohwa, 2023). This situation emphasizes that STEAM integration in Indonesia needs to be directed not only at academic achievement but also at the development of socio-cognitive competencies starting from the elementary school level.

Globally, STEAM has long been positioned as a transdisciplinary approach that combines knowledge acquisition with the development of social-cognitive skills. Developed countries such as South Korea, Japan, and the United States are beginning to utilize immersive technologies, including metaverses and VR/AR, to create collaborative learning experiences that enable students to develop empathy, communication, and problem-solving skills ( Chen, 2022). However, while numerous studies emphasize the potential of immersive technologies in enhancing learning motivation and engagement, research explicitly examining their impact on elementary school students’ social-cognitive skills is still scarce ( Lee, Shin & Gil, 2024). Thus, there is a significant opportunity to develop IMLE as an innovative medium that not only increases learning interest but also hones students’ social-cognitive competencies.

The literature confirms that the STEAM approach is inseparable from strengthening social-cognitive skills. This learning model is designed to familiarize students with collaborating to solve multidimensional challenges, thus demanding communication skills, emotional regulation, and collective reasoning ( García-Llamas et al., 2025; Suyundukova, 2025). If the social-cognitive dimension is ignored, STEAM learning tends to be reduced only to technical aspects and results, thus losing its essence as a transformative learning experience ( Suyundukova, 2025; Chang, Wang, & Ku, 2023). Therefore, strengthening the social-cognitive dimension through technology-based innovation is relevant to strengthening STEAM practices in elementary education.

Recent research emphasizes that social-cognitive skills are a key factor in the quality of collaborative learning ( Li, Wijaya, Chen, & Harahap, 2024; Chou, Hsieh, & Pan, 2024). In the context of science and technology projects, group work becomes more productive when members demonstrate empathy and can understand shared perspectives ( Chou, Hsieh, & Pan, 2024). Furthermore, students with good self-regulation tend to be more focused and open to others’ ideas ( Herro, Quigley, & Abimbade, 2021). This fact emphasizes that the success of STEAM implementation is largely determined by the mastery of social-cognitive skills ( Branan, 2025), the potential of which can be further strengthened through immersive learning experiences based on IMLE.

However, in reality, basic education in Indonesia still tends to emphasize solely cognitive aspects. Evaluation of learning outcomes relies heavily on test scores, while collaboration, communication, and conflict resolution skills are rarely used as formal indicators ( Guerrero et al., 2024). This hinders students’ socio-emotional development ( Raju, 2024). To bridge this gap, experts encourage the development of authentic assessments that balance academic achievement with socio-cognitive skills ( Akramova et al., 2024; Başaran & Bay, 2022). Furthermore, the limited availability of learning models specifically designed to develop socio-cognitive skills also poses a barrier ( Osterhaus, Koerber, & Sodian, 2017; Sun, You, & Zhou, 2023). In fact, the STEAM approach demands collaborative strategies, experiments, and active group dynamics that are in line with IMLE’s potential to create collaborative and interactive learning spaces ( Armi & Nisa, 2025).

Based on the description, two main gaps underlie this research. First, in Indonesia, the integration of social-cognitive skills in STEAM learning has not been systematically accommodated in the curriculum or assessment, resulting in suboptimal student development. Second, both at the national and global levels, research specifically examining the effectiveness of immersive technology particularly the Immersive Metaverse Learning Environment (IMLE) in supporting elementary school students’ social-cognitive skills is still very limited. Therefore, this research aims to examine the potential of IMLE as an innovative learning framework capable of improving students’ social-cognitive skills in the context of STEAM in elementary education.

### Development Immersive Metaverse Learning Environments (IMLEs) to enhance social-cognitive skills in STEAM learning in elementary schools in several countries

Immersive technologies, particularly Virtual Reality (VR) and Augmented Reality (AR), have been widely adopted in education as innovative media capable of delivering more realistic and interactive learning experiences. VR allows students to directly engage in simulated virtual environments, while AR enhances the real world by adding digital elements that aid understanding of abstract concepts. These two technologies collectively open up new opportunities to increase student engagement and support exploration-based learning ( Erawan, Mariana, & Suryanti, 2025; Wu, Liu, & Huang, 2022). Numerous empirical studies have shown that immersive technologies can enhance student learning motivation. Engaging visualizations and direct interaction foster greater interest and engagement in the learning process. Furthermore, VR/AR-based learning experiences shift students’ roles from passive recipients of information to active participants in knowledge construction. This shift implies a paradigm shift from teacher-centered to student-centered learning ( Wu, Liu, & Huang, 2022).

In the context of collaboration, immersive technology provides a space for students to work together in a digital environment that resembles the real world. Several studies have shown that the application of VR in collaborative science experiments can improve students’ communication and cooperation skills. This confirms that the benefits of immersive technology are not limited to cognitive aspects but also include strengthening broader social-cognitive skills ( Lampropoulos & Kinshuk, 2024). Despite its great potential, the implementation of immersive technology in elementary schools faces various challenges. The main obstacle is limited access to devices and supporting infrastructure, as many schools do not yet have the resources to provide VR/AR equipment. Furthermore, physical challenges such as dizziness or fatigue due to device use are other factors that need to be considered ( Asoodar, Janesarvatan, & de Jong, 2024).

Another challenge is the low technological literacy among elementary school teachers. Many educators lack the pedagogical and technical skills to integrate immersive technology into everyday learning practices. As a result, this technology is often used in a limited manner or merely as entertainment, rather than as an integral part of the learning strategy. This situation emphasizes the need for comprehensive training support and clear learning models to optimize the use of immersive technology ( Tao, Cukurova, & Song, 2025). A literature review shows that the Immersive *Metaverse Learning Environment* (IMLE) has become a rapidly growing topic globally, particularly in relation to STEAM-based learning. However, research remains unequal; publications largely originate from developed countries such as the United States, China, South Korea, Japan, and European countries, while contributions from developing countries, including Indonesia, are relatively minimal ( Shen et al., 2023; Sripan & Jeerapattanatorn, 2025). Furthermore, existing research focuses more on the learning experience, motivation, and exploration of VR/AR technology, rather than on in-depth analysis of the effectiveness of IMLE in developing elementary school students’ social-cognitive skills ( Rahman et al., 2024; Wang et al., 2023). Yet, social-cognitive dimensions such as collaboration, communication, empathy, and self-regulation are key elements of 21st-century STEAM learning.

Table 1 shows that research related to IMLE is still dominated by developed countries, with the United States and China being the largest contributors. Meanwhile, Indonesia is just beginning to explore the potential of VR/AR in primary education, but not many have explicitly studied the effectiveness of IMLE for social-cognitive skills. This suggests that research *is significant*, namely the need for empirical research focused on the implementation of IMLE in the context of Indonesian elementary schools, by measuring its impact on students’ social-cognitive skills in STEAM learning. Thus, this research has the potential to make an important contribution in bridging the gap in global literature while enriching elementary education practices in Indonesia.

**Table 1.** Summary of IMLE research in selected countries (2022–2025).

Country	Main study focus	Reference source
United States of America	Metaverse development for student collaboration & engagement	Wang et al. (2023); Shen et al. (2023)
China	VR/AR-based immersive technology innovation for science learning	Shen et al. (2023)
South Korea	Integration of IMLE in STEAM-based elementary education	Sripan & Jeerapattanatorn (2025)
Japan	A small experiment on using VR/AR for collaborative learning	Sripan & Jeerapattanatorn (2025)
European countries (combined)	Theoretical review & case study of IMLE usage in elementary grades	Shen et al. (2023)
Indonesia	Limited use of VR/AR, focus on learning motivation	Rahman et al. (2024)

## Method

### Research design

This research employs a pragmatist paradigm, which emphasizes selecting methods appropriate to the research objectives and problems. This paradigm is relevant because the research not only quantitatively tests the effectiveness of the Immersive Metaverse Learning Environment (IMLE) but also examines experiences and contextual factors through a qualitative approach. The chosen design is a mixed-methods explanatory sequential design. The first stage involves collecting quantitative data to measure the impact of IMLE on elementary school students’ social-cognitive skills. The next stage, a qualitative phase, is conducted to explain the quantitative findings through interviews and observations. The justification for choosing this design is that the primary focus of the research is on testing the effectiveness of the intervention, while qualitative data is needed to understand the mechanisms and experiences underlying the quantitative results. Thus, the explanatory sequential design strikes a balance between the rigor of statistical analysis and the depth of contextual understanding, resulting in more comprehensive findings.

### Participants

The participants of this study consisted of 100 elementary school students and accompanying teachers, who were selected using purposive sampling techniques. The research location includes several elementary schools in city/district X, which have adequate technological facilities for implementation. *Immersive Metaverse Learning Environment* (IMLE). Student demographics included ages 9–11, male and female, and varied academic backgrounds. The teachers involved were educators who taught STEAM subjects and had at least one year of experience teaching in related classes. The justification for selecting 100 students was based on methodological and practical considerations. This number was deemed sufficient for quantitative analysis with a 95% confidence level and a 5% margin of error. This also allows for in-depth qualitative data collection through interviews and observations. This sample size is also consistent with previous research examining immersive technology-based interventions in elementary schools. The demographics of the study participants are shown in Table 2.

**Table 2.** Demographics of research participants.

Category	Sub-category	Number (n)	Percentage (%)
Student	Age 9 years	70	70%
	Age 10 years	30	30%
	Man	36	36%
	Woman	64	64%
Accompanying teacher	Total teachers	5	100%

### Participant informed consent

Prior to conducting the study, the researcher provided a complete explanation to all participants regarding the purpose, benefits, and procedures of the study, including how data was collected and how their identities would be kept confidential. This study involved students and teachers in an elementary school. To maintain research ethics, written informed consent was obtained from teachers, while verbal assent from students was given after receiving an explanation in simple, age-appropriate language. Verbal consent was chosen for students because they are still in the developmental stage of childhood, and direct communication is considered more effective in explaining their rights and involvement in the research. The researcher ensured that all participation was voluntary and free from any pressure from any party.

### Data collection

Data collection in this study was carried out in stages according to the design *explanatory sequential.* Quantitative data were collected first through a social-cognitive skills test and questionnaires administered to 100 participating students, each session lasting approximately 60 minutes. Next, qualitative data were collected to explain the quantitative findings, including in-depth interviews with 10 students and 5 accompanying teachers for 30–45 minutes per participant, focus group discussions (FGDs) with all teachers for approximately 60 minutes, and participatory classroom observations for approximately 90 minutes per learning session to record social interactions, collaboration, and student engagement during the use of the *Immersive Metaverse Learning Environment* (IMLE). This study was conducted over 10 weeks, starting with instrument preparation and school coordination (weeks 1–2), intervention implementation and quantitative data collection (weeks 3–6), and qualitative data collection, initial analysis, and integration of quantitative-qualitative results (weeks 7–10). This procedure was designed to ensure data validity and reliability, as well as to gain a deeper understanding of the effectiveness of IMLE in improving elementary school students’ social-cognitive skills.

### Data analysis

Data analysis in this study was conducted using a mixed methods approach following an explanatory sequential design. Quantitative data were analyzed using descriptive and inferential statistical techniques to measure the effect of the Immersive Metaverse Learning Environment (IMLE) intervention on students’ social-cognitive skills, while qualitative data were used to explain the quantitative findings and understand the mechanisms of social-cognitive behavior change.

To enhance the validity of the findings, the study employed operational triangulation by comparing information from various sources, namely quantitative tests, questionnaires, interviews, focus group discussions (FGDs), and classroom observations, to obtain a comprehensive understanding of the effectiveness of IMLE. All interviews and FGDs were transcribed verbatim, and transcript verification was conducted through re-examination of the original recordings and confirmation with participants (member checking) to ensure the accuracy of interpretation. Furthermore, the study employed an audit trail, where all documents, field notes, transcripts, codes, and methodological decisions were systematically recorded, enabling traceability of the analysis process and assessing the consistency of data interpretation.

Data integrity was maintained through encrypted digital storage, regular backups, and anonymized participant management, as well as the application of ethical research principles. Analysis was conducted sequentially: quantitative data were analyzed to identify changes in social-cognitive skills using paired sample t-tests and descriptive analyses, while qualitative data were analyzed using thematic analysis to identify themes that explained the quantitative results. The results of both approaches were then integrated to provide a comprehensive overview of the effectiveness of IMLE in supporting students’ social-cognitive skill development in STEAM learning in elementary schools.

## Results

Social-cognitive skills were measured before and after the context-based learning intervention. *Immersive Metaverse Learning Environment* (IMLE). Descriptive data, including mean, standard deviation, and mode, are presented in Table 3. Quantitative results indicate significant improvements in students’ collaboration, empathy, communication, and self-regulation skills following the IMLE intervention. These findings are reinforced by qualitative data from interviews, focus group discussions, and classroom observations, which revealed that students were more active in collaborative problem-solving, demonstrated greater empathy toward peers, and were better able to self-regulate during STEAM activities. This integration of quantitative and qualitative evidence provides a strong explanation for how and why IMLE can improve social-cognitive skills. Theoretically, these results align with social-cognitive learning theory, which emphasizes the importance of interaction, modeling, and feedback in learning environments that support the development of social-cognitive competencies ( Bandura, 1986). This study makes a novel contribution by demonstrating that IMLE can operationalize these theoretical principles in a concrete immersive learning context for elementary school students, offering an innovative approach to bridging STEAM content mastery with social-cognitive skill development.

**Table 3.** Descriptive statistics of social-cognitive skills.

Measurement	Mean	Standard deviation	Mode	Minimum	Maximum
Pre-test	68.28	8.10	74	51	84
Post-test	80.01	7.05	89	75	98

Table 3 presents the descriptive analysis results, indicating a clear improvement in students’ social-cognitive skills following participation in IMLE-based learning. The average student score on the post-test was higher than on the pre-test, with a relatively more consistent distribution of scores, as indicated by a decrease in standard deviation. The mode also shifted toward higher scores, suggesting that more students achieved optimal achievement after the treatment. Furthermore, the minimum and maximum score ranges on the post-test showed a positive shift, with the lowest score increasing and the highest score also improving compared to before the treatment. Overall, these findings indicate that the use of IMLE in STEAM learning contributes significantly to improving students’ social-cognitive skills. To determine the significance of the difference in social-cognitive skill scores before and after IMLE-based learning, a paired-samples t-test was conducted. The results of the analysis are presented in Table 4.

**Table 4.** Paired-samples t-test results: Pre-test and Post-test.

Variables	Mean difference	t	df	Sig. (2-tailed)
Social-Cognitive (Pre-Post)	-17.37	19.82	149	0.000

Table 4 explains the results of the paired-samples t-test, which showed a significant difference between the pre-test and post-test scores for students’ social-cognitive skills. The mean difference showed a consistent increase, while the statistical test results showed that the difference was highly statistically significant. This indicates that the implementation of IMLE in STEAM learning has a significant positive impact on the development of elementary school students’ social-cognitive skills. In other words, the changes that occurred were not coincidental but rather a direct result of the IMLE-based learning intervention. Based on analysis of teacher interviews, student focus group discussions, and field observations.

Based on the quantitative analysis results that showed significant improvements in students’ social-cognitive skills, the next step was to present qualitative findings that provided an in-depth understanding of the mechanisms, experiences, and contexts underlying these changes. Qualitative data from teacher interviews, student group discussions, and field observations were used to interpret how IMLE facilitated social interaction, collaboration, empathy, and self-regulation in STEAM activities. This approach allowed researchers not only to observe improvements in scores but also to understand the learning processes that occurred, allowing quantitative findings to be explained from the perspective of students’ and teachers lived experiences. To understand how the IMLE intervention affected students’ social-cognitive behavior, qualitative data were used to complement and explain the quantitative results. Qualitative data were explained based on two sub-themes: first, Improving Student Interaction and Collaboration in IMLE Learning; and second, Developing Empathy and the Ability to Understand Other Perspectives.

### Improving student interaction and collaboration in IMLE learning

The use of Immersive Metaverse Learning Environment (IMLE) technology is currently attracting the attention of many educators due to its ability to provide a learning experience that is close to the real world. Through a combination of virtual reality, augmented reality, and interactive digital spaces, students can explore learning concepts in a more concrete and participatory manner. However, various findings in the field show that the success of IMLE implementation depends not only on the sophistication of the technology, but also on how teachers are able to foster interaction and collaboration among students in this virtual environment. Teachers, who act not only as material deliverers but also as social facilitators, play a crucial role in fostering meaningful learning engagement. Observations in several schools that have implemented IMLE show that students tend to be more enthusiastic and actively participate when they are faced with a three-dimensional learning space with personal avatars. In this situation, students feel freer to express their ideas and interact without feeling awkward. However, the challenge that arises is how teachers can direct these interactions so that they are oriented towards academic collaboration, rather than simply visual exploration. Some teachers overcome this by designing group mission-based activities that require a balance of roles and responsibilities for each member.

To understand how the Immersive Metaverse Learning Environment (IMLE) intervention impacts students’ social-cognitive behavior, qualitative data were used to complement and explain the quantitative results. Observations during IMLE-based learning activities showed that students tended to be more actively engaged in group discussions and exhibited more focused collaboration patterns compared to conventional learning. They not only worked simultaneously but also developed collaborative dynamics that complemented and adapted to the group’s needs. For example, in a digital bridge-building simulation activity, students naturally divided roles: one designed the structure, another calculated strength and balance, and another tested the design’s durability. This division of tasks emerged from the students’ own initiative without direct guidance from the teacher, demonstrating their ability in collective decision-making and responsibility management. Furthermore, these interactions led to critical discussions, where students provided feedback and considered their peers’ suggestions before making final decisions, indicating improved reflective thinking skills and collaborative problem-solving abilities. This confirms that IMLE not only encourages active engagement but also stimulates the development of more complex social-cognitive competencies, including empathy, effective communication, and shared responsibility within an immersive learning context.

Teachers also noted that students who were typically passive in real classes became more vocal in the metaverse environment, confidently expressing ideas and actively participating in group decision-making. This demonstrates IMLE’s potential to create a more participatory learning environment and encourage greater student engagement. Student interview descriptions support these findings, as one student stated:

*“I’ve become more confident asking my friends if I don’t understand something. It feels like we’re playing together, so I’m not embarrassed to ask for help. In the past, I used to feel nervous and worried that people might think I wasn’t smart enough when I didn’t get something right away. But now, I realize that learning is more fun and meaningful when we do it together. My friends are always kind and supportive, and they often have different ways of explaining things that make it easier for me to understand. When we discuss or solve problems as a group, it feels more like teamwork than studying alone. I’ve learned that asking questions doesn’t make you weak; it shows you’re curious and willing to learn. This change has made me more active and engaged in class, and I feel more connected with my friends because we help each other grow.”* (Student A, FGD).

This statement suggests that the IMLE learning experience fosters a sense of psychological safety, making students more willing to take social risks by asking questions and expressing opinions. Teacher interviews also corroborate this finding:

*“At IMLE, students seem more open to sharing ideas. Even students who are usually quiet are now the ones who offer the most suggestions.”* (Teacher B, individual interview).

The integration of observation and interview results indicates that IMLE catalyzes enhancing students’ collaborative interactions. This immersive technology not only provides a fun learning experience but also provides a safe space to experiment with social roles, strengthen communication skills, and foster mutual trust among students. Therefore, enhancing collaborative interactions through IMLE is an important aspect in developing social-cognitive skills in the context of STEAM learning in elementary schools. Theoretically, these findings align with theory Bandura’s (1986) social-cognitive learning **,** which emphasizes the importance of interaction, modeling, and feedback in building social-cognitive competencies. In other words, the increase in students’ quantitative scores can be explained through the observed interaction and collaboration mechanisms in IMLE, so that the qualitative data provide a context that strengthens the validity of the quantitative findings while supporting social-cognitive theory. Teachers’ experiences in managing IMLE learning demonstrate a significant shift in their roles. They are no longer information centers, but rather learning experience designers and social interaction facilitators. Successful teachers typically prepare contextual learning scenarios, group students heterogeneously, and utilize real-time feedback features to monitor collaboration in the virtual world.

### Developing empathy and the ability to understand other perspectives

In addition to increasing collaboration, the application of IMLE in STEAM learning also encourages the development of empathy and the ability to take the perspective of others ( *perspective-taking *). During the activity, students were more sensitive to the needs of their groupmates. When a student encountered technical difficulties or fell behind in completing a mission, their peers immediately provided support through verbal encouragement and practical assistance. Some students even paused their activities to explain the steps to solve the problem, demonstrating concern for the learning needs of others. The use of avatars in IMLE also plays a crucial role in encouraging students to express their ideas without fear of ridicule or criticism. This psychological distance allows students to be more open in sharing ideas, responding to peers’ opinions, and asking questions, creating an inclusive, collaborative atmosphere where every contribution is valued. Teachers also observe that students are increasingly accustomed to listening to their peers’ perspectives before making decisions together. They are more likely to accept differences of opinion, seek to understand the reasons behind them, and then negotiate mutually agreeable solutions. Student interview findings support this observation, with one student stating:

*“If a friend is left behind, I want to help explain so we can complete the mission together. It feels good when everyone succeeds.”* (Student B, individual interview).

Teacher interviews added a similar perspective:

*“I’ve seen students become more patient in listening to each other’s opinions, and some even try to understand the reasons behind differing ideas. This is rarely seen in a real classroom.”* (Teacher C, individual interview).

These results confirm that IMLE provides a space for students to practice taking others’ perspectives, a crucial social skill in elementary school. The integration of observation and interview results indicates that IMLE plays a significant role in fostering empathy and the ability to understand peers’ perspectives. Thus, learning experiences in the metaverse not only strengthen the cognitive aspects of STEAM but also build a foundation for social skills essential for child development.

This finding is consistent with social-cognitive learning theory **,** which emphasizes the role of social interaction, peer observation, and active learning experiences in developing social skills. Thus, the integration of quantitative and qualitative results indicates that IMLE not only enhances the academic aspects of STEAM but also provides a space for the practice of empathy and social perspective, bridging theory with students’ real-life learning experiences.

## Discussion

The findings of this study indicate that the implementation Immersive Metaverse Learning Environment (IMLE) in STEAM learning significantly improved elementary school students’ social-cognitive skills. Quantitative analysis indicated an increase in mean scores after treatment, significantly different from the baseline. This increase was also supported by qualitative results showing growth in collaborative dynamics, empathy, and perspective-taking during learning activities in the metaverse world. This aligns with the findings of Abukhousa, El-Tahawy, & Atif (2023), who stated that metaverse-based immersive environments provide a more participatory learning experience because they can reduce students’ psychological barriers to interaction. Thus, IMLE has been shown to function not only as a STEAM learning medium but also as a vehicle for developing essential 21st-century social skills ( Căpățînă et al., 2024; Jurado-Vásquez, Ultreras-Rodríguez, & Herrera, 2024).

Furthermore, the increased collaborative interactions observed in this study confirm that IMLE is capable of creating a learning ecosystem that encourages active student engagement. The phenomenon of typically passive students becoming more vocal in the metaverse world indicates that immersive technology can overcome traditional barriers to classroom participation ( Lee, Shin, & Gil, 2024). Similar research by Best et al. (2024) also confirmed that the use of virtual environments can broaden student participation by creating a sense of social safety and increasing confidence in sharing ideas. This is particularly important in the STEAM context, as project-based learning demands cross-disciplinary communication, coordination, and collaboration skills. In other words, IMLE functions as a facilitator of more balanced social interactions among students ( Youssef, Malak, & Bayoumy, 2025; Loia, Capolupo, & Adinolfi, 2025).

The results of this study also show an increase in empathy and perspective-taking, which is a core aspect of social-cognitive skills. Students not only helped peers experiencing difficulties but also demonstrated a willingness to listen to others’ perspectives before making group decisions. This supports the findings of Tan, Chye, & Teng (2022), who emphasized that collaborative learning is effective in improving empathy and interpersonal skills when facilitated by technology that can minimize communication barriers. Furthermore, the use of avatars in IMLE has been shown to help students feel more emotionally secure, consistent with a study by Hidayanthi, Siregar, Siregar & Siregar (2024) who found that virtual representations can reduce social anxiety and encourage affective engagement.

Furthermore, the integration of quantitative and qualitative results in this study indicates that improvements in social-cognitive skills occur not only at the final level but also throughout the learning process. The significant differences between the pre-test and post-test align with the dynamics of collaboration, empathy, and social perspective observed in the field. This integration strengthens the validity of the findings and supports the view of Lee, Shin, & Gil (2024) that the social-cognitive approach *mixed-methods *effective in technology-based educational research because it can capture the complexity of change in both outcomes and processes. Thus, the results of this study provide strong empirical evidence regarding the effectiveness of IMLE in STEAM learning in elementary schools ( Brady, Leonard & Choisdealbha, 2024; Hidayanthi, Siregar, Siregar & Siregar, 2024).

An important addition to this finding is that this study is not only consistent with previous research but also extends it by demonstrating how IMLE can specifically operationalize Bandura’s (1986) social-cognitive learning theory in the context of STEAM in elementary schools. Thus, this study not only supports previous literature but also provides a new contribution in the form of integrative evidence that connects quantitative, qualitative, and theoretical data in explaining the improvement of social-cognitive skills through immersive technology.

The implication of this research is the importance of integrating metaverse technology into the elementary education curriculum to strengthen 21st-century skills, particularly in the social-cognitive aspect. This will enhance collaboration, empathy, and interpersonal skills. *perspective-taking* The findings in this study indicate that IMLE has potential as an innovative learning strategy that supports learning objectives. *Education for Sustainable Development* (ESD). This aligns with UNESCO’s findings United Nations Educational, Scientific and Cultural Organization (2020) which emphasize that future education must integrate digital literacy, collaboration, and social skills to address global challenges. Therefore, IMLE is not just a learning technology but also a pedagogical platform capable of connecting cognitive and social competencies in an integrated manner, thereby enriching students’ learning experiences in the digital age.

## Conclusion

Based on the results of this study, it can be concluded that the application of technology, the Immersive *Metaverse Learning Environment* (IMLE), has been proven effective in improving elementary school students’ social-cognitive skills in STEAM learning. Quantitative results show significant improvements in collaboration, empathy, communication, and social decision-making. Meanwhile, qualitative results show that students are more active, confident, and inclusive in interacting in virtual environments. The integration of IMLE with STEAM learning also has a positive impact on student motivation and overall learning outcomes. The uniqueness of this study lies in its application in the context of elementary education in Indonesia, a field rarely researched both nationally and globally. Overall, the findings align with previous studies that confirm that immersive technology can create participatory learning experiences and support the development of social skills. However, this study provides an extension by demonstrating how IMLE can specifically be operationalized to integrate social-cognitive skills into STEAM learning in elementary schools, a context that has not been widely explored in the international literature.

## Ethics and consent

This research has obtained ethical approval from the Research Ethics Committee of the Institute for Research and Community Service, Universitas PGRI Adi Buana Surabaya, Indonesia, with certificate number 11.1/Ad/RE/LPPM/V/2025, dated June 20, 2025. The research protocol was approved after an ethical review process that included participant protection, data confidentiality, informed consent, and adherence to national and international research ethics principles. Written informed consent was obtained from teachers, while verbal assent was provided by students after receiving explanations in age-appropriate language. The researchers ensured that all procedures were carried out in accordance with the WMA Declaration of Helsinki – Ethical Principles for Medical Research Involving Human Participants, to ensure research integrity, safety, and respect for the rights of each participant.

## Data Availability

Figshare: Immersive Metaverse Learning Environment (IMLE) to Improve Social-Cognitive Skills in STEAM Learning in Elementary Schools. DOI: https://figshare.com/s/ede7789f2f88d13f6671 ( Rachmadtullah et al., 2025a) Figshare: Journal contribution. Doi: https://figshare.com/s/297d32d1e497824f25b1 ( Rachmadtullah et al., 2025b) Data are available under the terms of the Creative Commons Attribution 4.0 International license (CC-BY 4.0)
