# Supplementary material for: Immersive Metaverse Learning Environment (IMLE) to Improve Social-Cognitive Skills in STEAM Learning in Elementary Schools
Source: F1000Res. 2025 Dec 22;14:1428. [Version 1] doi: 10.12688/f1000research.172295.1 (PMC13178625; doi:10.12688/f1000research.172295.1)
Supplement: Peer review history

# Reviewer report: Reviewer response for version 1

**DOI:** 10.5256/f1000research.190011.r462139

**Published:** 2026-03-05

**Authors:** Nurul Mahruzah Yulia

**Affiliations:** 1 Nahdlatul Ulama Sunan Giri University, Bojonegoro, East Java, Indonesia

1. Summary of the Article

 This article investigates the effectiveness of an **Immersive Metaverse Learning Environment (IMLE)** in improving **social-cognitive skills in STEAM learning among elementary school students**. The authors argue that 21st-century education requires not only cognitive development but also social-cognitive competencies such as collaboration, empathy, communication, and self-regulation. The study adopts a **mixed-methods explanatory sequential design**, where quantitative data are collected first to examine the impact of IMLE on students’ social-cognitive skills, followed by qualitative data to explore student and teacher experiences

 The research involved **100 elementary school students and several teachers in Indonesia**, with data collected through pre-tests, post-tests, questionnaires, interviews, focus group discussions, and classroom observations. Quantitative analysis using **paired-sample t-tests** indicated a significant increase in students’ social-cognitive skills after participating in IMLE-based learning activities. Qualitative findings further suggested that immersive learning environments facilitated collaboration, empathy, and perspective-taking during STEAM activities. 

 The authors conclude that IMLE can serve as an innovative learning framework that supports the development of both cognitive and social competencies in elementary school education

 2. Overall Evaluation

 The article addresses an **important and emerging topic**, namely the use of immersive technologies and metaverse environments in STEAM education. The integration of **social-cognitive theory with immersive learning environments**represents a promising direction for research in educational technology.

 The study demonstrates several strengths, particularly the integration of **quantitative and qualitative data**, the relevance of the research context, and the potential pedagogical implications for elementary education.

 However, several sections require **substantial revision** to ensure the article meets the standards of scientific rigor expected in international academic journals. The most important concerns relate to the **clarity of the research design, methodological transparency, and the interpretation of results**.

 3. Major Strengths of the Article

 3.1 Relevance of the Topic

 The research addresses a timely issue in educational technology, particularly the use of immersive learning environments in STEAM education.

 3.2 Integration of Social-Cognitive Theory

 The article appropriately connects its findings with **Bandura’s social-cognitive theory**, which provides a theoretical explanation for collaborative learning and behavioral development in immersive environments.

 3.3 Mixed-Methods Approach

 The use of both **quantitative and qualitative data** allows the authors to examine not only the outcomes of the intervention but also the learning processes experienced by students.

 3.4 Practical Educational Implications

 The study provides useful insights for teachers and educational institutions regarding the use of immersive technologies to support collaborative learning.

 Recommendation

 The authors must:

- clarify the **exact number of participants**
- ensure that the **sample size is consistent across all sections**
- explain how participants were allocated to the study.

This issue must be corrected for the study to be scientifically sound.

 Important information is missing, including:

- the structure of the IMLE learning activities
- duration of each learning session
- technological platform used
- specific STEAM tasks performed by students
- teacher roles during the intervention

Is the work clearly and accurately presented and does it cite the current literature?

Yes

If applicable, is the statistical analysis and its interpretation appropriate?

Yes

Are all the source data underlying the results available to ensure full reproducibility?

Partly

Is the study design appropriate and is the work technically sound?

Yes

Are the conclusions drawn adequately supported by the results?

Yes

Are sufficient details of methods and analysis provided to allow replication by others?

Partly

Reviewer Expertise:

STEAM Learning; Elementary School; Gamifikasi; Scientific Literacy; Digital Learning

I confirm that I have read this submission and believe that I have an appropriate level of expertise to confirm that it is of an acceptable scientific standard.

# Reviewer report: Reviewer response for version 1

**DOI:** 10.5256/f1000research.190011.r463479

**Published:** 2026-03-05

**Authors:** Raziska Ibrahimi

**Affiliations:** 1 Universitas Bina Bangsa Getsempena, Banda Aceh, Indonesia

Conceptually, this approach aligns with social constructivism theory, which emphasizes the importance of interaction, collaboration, and authentic experiences in the learning process. Immersive environments enable students to construct understanding through contextual simulations that are difficult to achieve in conventional classrooms. With the right design, IMLE has the potential to enhance problem-solving skills, empathy, perspective-taking, and self-regulation, which are essential components of social-cognitive competency.

 In terms of novelty, this research is relevant to the transformation of education based on cutting-edge technology, particularly in utilizing the metaverse ecosystem as a pedagogical medium, not simply as a virtual space. If developed with a comprehensive evaluation model, this research could serve as an important reference for the development of immersive technology-based STEAM curricula at the elementary level.

 The practical implications are also quite broad. The results of this study have the potential to encourage schools and policymakers to design more systematic technology integration strategies, including aspects of teacher readiness, infrastructure, and strengthening students' digital literacy. Thus, this research not only contributes theoretically but also has strategic implications for the development of innovative and sustainable learning in elementary schools.

Is the work clearly and accurately presented and does it cite the current literature?

Yes

If applicable, is the statistical analysis and its interpretation appropriate?

Yes

Are all the source data underlying the results available to ensure full reproducibility?

Yes

Is the study design appropriate and is the work technically sound?

Yes

Are the conclusions drawn adequately supported by the results?

Yes

Are sufficient details of methods and analysis provided to allow replication by others?

Yes

Reviewer Expertise:

elementary education

I confirm that I have read this submission and believe that I have an appropriate level of expertise to confirm that it is of an acceptable scientific standard.

# Reviewer report: Reviewer response for version 1

**DOI:** 10.5256/f1000research.190011.r462140

**Published:** 2026-03-04

**Authors:** Ary Kiswanto Kenedi

**Affiliations:** 1 Universitas Samudra, Langsa, Indonesia

This manuscript is relevant and makes a clear contribution to the integration of the Immersive Metaverse Learning Environment (IMLE) in STEAM learning at the elementary school level to strengthen students’ social-cognitive skills. The mixed-methods design is appropriate for the research objectives, and the quantitative findings are well supported by qualitative evidence. I recommend **acceptance with minor revisions**, particularly to ensure consistency in the reported sample size and degrees of freedom (df), to include effect size in the statistical analysis, and to further clarify the measurement instruments and details of the intervention to improve the study’s replicability.

Is the work clearly and accurately presented and does it cite the current literature?

Yes

If applicable, is the statistical analysis and its interpretation appropriate?

Yes

Are all the source data underlying the results available to ensure full reproducibility?

Yes

Is the study design appropriate and is the work technically sound?

Yes

Are the conclusions drawn adequately supported by the results?

Yes

Are sufficient details of methods and analysis provided to allow replication by others?

Yes

Reviewer Expertise:

The study addresses a relevant and emerging topic by examining the role of Immersive Metaverse Learning Environments (IMLE) in improving social-cognitive skills in elementary STEAM learning. The research design is appropriate and the findings are valuable. However, several issues should be clarified, including inconsistencies in the reported sample size and statistical reporting (df), the inclusion of effect size in the analysis, and clearer descriptions of the instruments and intervention procedures. Therefore, I recommend approval with reservations pending minor revisions.

I confirm that I have read this submission and believe that I have an appropriate level of expertise to confirm that it is of an acceptable scientific standard, however I have significant reservations, as outlined above.
